# Supplementary material for: Mechanisms governing the pioneering and redistribution capabilities of the non-classical pioneer PU.1
Source: Nat Commun. 2020 Jan 21;11:402. doi: 10.1038/s41467-019-13960-2 (PMC6972792; doi:10.1038/s41467-019-13960-2)
Supplement: Supplementary file 7 — Source data [file 41467_2019_13960_MOESM7_ESM.zip › Source_Data/FigureS3/FigureS3J_MotifScanOutput/homerResults/motif4.similar.html]

motif4

## Information for motif4

C
A
T
G
A
G
C
T
G
C
A
T
C
G
T
A
T
G
C
A
C
G
A
T
T
C
A
G
C
G
A
T
C
A
G
T
A
C
G
T
C
T
G
A
G
T
C
A
  
Reverse Opposite:  

C
A
G
T
G
A
C
T
C
G
T
A
G
T
C
A
G
C
T
A
A
G
T
C
C
G
T
A
A
C
G
T
A
C
G
T
C
G
T
A
T
C
G
A
G
T
A
C
  

|  |  |
| --- | --- |
| p-value: | 1e-222 |
| log p-value: | -5.122e+02 |
| Information Content per bp: | 1.703 |
| Number of Target Sequences with motif | 1509.0 |
| Percentage of Target Sequences with motif | 11.43% |
| Number of Background Sequences with motif | 1273.0 |
| Percentage of Background Sequences with motif | 3.49% |
| Average Position of motif in Targets | 368.1 +/- 308.6bp |
| Average Position of motif in Background | 328.9 +/- 193.0bp |
| Strand Bias (log2 ratio + to - strand density) | 0.0 |
| Multiplicity (# of sites on avg that occur together) | 1.08 |
| Motif File: | file (matrix) reverse opposite |

### Similar de novo motifs found

|  |  |  |  |  |  |  |  |
| --- | --- | --- | --- | --- | --- | --- | --- |
| Rank | Match Score | Redundant Motif | P-value | log P-value | % of Targets | % of Background | Motif file |
| 1 | 0.883 | C A T G A G C T G C A T C G T A T G C A G C A T C T A G C G T A G C A T G A C T G C T A T C G A G T A C | 1e-211 | -486.227711 | 10.36% | 3.03% | motif file (matrix) |
| 2 | 0.781 | C T G A C T G A C G A T A T C G C G T A A G C T C G A T C T G A T C G A A T G C | 1e-200 | -460.857730 | 36.52% | 22.71% | motif file (matrix) |
| 3 | 0.884 | C G T A C A T G G A C T G C A T T G C A G T C A G C A T A C T G C G T A C A G T G A C T G T C A C T G A G T A C | 1e-186 | -429.789683 | 8.09% | 2.09% | motif file (matrix) |
| 4 | 0.763 | T A C G G A C T G C A T C G T A C T G A A G C T T G A C G C T A C A G T | 1e-57 | -131.388282 | 21.80% | 15.55% | motif file (matrix) |
